# Rapid diagnostics for point-of-care quantification of soluble transferrin receptor

**DOI:** 10.1016/j.ebiom.2019.03.017

**Published:** 2019-03-16

**Authors:** Balaji Srinivasan, Julia L. Finkelstein, Dakota O’Dell, David Erickson, Saurabh Mehta

**Affiliations:** a Division of Nutritional Sciences, Cornell University, Ithaca, NY, USA; b Institute for Nutritional Sciences, Global Health, and Technology (INSiGHT), Cornell University, Ithaca, NY, USA; c VitaMe Technologies Inc., Ithaca, NY, USA; d Sibley School of Mechanical and Aerospace Engineering, Cornell University, Ithaca, NY, USA

**Keywords:** Iron deficiency, Anaemia, Lateral flow immunoassay, Soluble transferrin receptor, Point-of-care testing, Portable diagnostics

## Abstract

**Background:**

Iron deficiency (ID) and anaemia are major health concerns, particularly in young children. Screening for ID based on haemoglobin (Hb) concentration alone has been shown to lack sensitivity and specificity. The American Academy of Pediatrics (AAP) recommends soluble transferrin receptor (sTfR) as a promising approach to screen for iron deficiency. However, in most settings, assessment of iron status requires access to centralized laboratories. There is an urgent need for rapid, sensitive, and affordable diagnostics for sTfR at the point-of-care.

**Methods:**

An immunochromatographic assay-based point-of-care screening device was developed for rapid quantification of sTfR from a drop of serum within a few minutes. Performance optimization of the assay was done in sTfR-spiked buffer and commercially available sTfR calibrator, followed by a small-scale proof-of-concept validation with archived serum samples.

**Findings:**

On preliminary testing with archived serum samples and comparison with Ramco ELISA, a correlation of 0.93 (P < 0.0001) was observed, demonstrating its potential for point-of-care assessment of iron status.

**Interpretation:**

The analytical performance of the point-of-care sTfR screening device indicates the potential for application in home-use test kits and field settings, especially in low- and middle-income settings. An added advantage of sTfR quantification in combination with our previously reported serum ferritin diagnostics is in integration of Cook's equation as a quantitative and minimally-invasive indicator of total body iron stores.

**Fund:**

Thrasher Research Fund (Early Career Award #13379), NIH R03 EB 023190, NSF grant #1343058, and Nutrition International (project #10-8007-CORNE-01).

**Research in context**

**Evidence before this study**

Iron deficiency and anaemia are urgent global health problems, particularly among young children. Screening for iron deficiency by determination of haemoglobin (Hb) concentrations alone at 12 months of age has been shown to lack sensitivity and specificity. The American Academy of Pediatrics (AAP) has identified sTfR screening as a promising approach for ID and supports the development of sTfR standards for infants. Quantification of sTfR can enable early detection of ID in individuals who are not yet anaemic. At present, in most settings, assessment of iron status requires access to centralized laboratories, uses blood obtained by venepuncture, and is time-consuming and often not affordable. There is an urgent need for rapid and sensitive diagnostics for ID at the point-of-care.

**Added value of this study**

At present, there are no commercially available portable point-of-care method for assessment of iron status. The point-of-care screening device for quantification of sTfR reported in this study has the potential to make quantification of sTfR more widely accessible to paediatricians and other health care providers. An added advantage of sTfR quantification in combination with our previously reported serum ferritin test is in integration of Cook's equation as a quantitative and minimally-invasive indicator of total body iron stores.

**Implications of all the available evidence**

Our preliminary findings based on small-scale proof-of-concept validation highlight the clinical potential of the point-of-care sTfR assessment on a mobile platform as a screening tool for ID at low-cost in home-use diagnostics, clinics, and for use in resource-limited and field settings.

Alt-text: Unlabelled Box

## 1 Introduction

Iron is the most common nutritional deficiency among infants and children, with an estimated prevalence of 3–80% [1], and is associated with long-term detrimental effects on neurodevelopment [2,3] which may be irreversible. The American Academy of Pediatrics (AAP) recommends universal screening for iron deficiency by determination of haemoglobin (Hb) concentrations with a cut-off at <11·0 g/dL at 12 months of age. However, this approach of using Hb concentration as a measure of iron status has been shown to have low sensitivity and specificity [4]. Among the biomarkers recommended by the World Health Organization (WHO) for assessment of iron status, a combination of ferritin and soluble transferrin receptor (sTfR) levels reflects the full spectrum of iron status from normal iron stores to tissue iron deficiency [5,6]. Assessment of sTfR is particularly important in contexts where iron requirements and cellular uptake are increased for growth and development, such as during pregnancy [7,8], infancy [9,10], and early childhood [11,12]. Elevated sTfR concentrations are observed in iron-deficient erythropoiesis, with levels rising even prior to decline in haemoglobin concentrations. Quantification of sTfR can therefore be applied for early detection of ID in individuals who are not yet anaemic. The AAP supports the development of sTfR standards [4] for use of sTfR diagnostics in screening for ID in infants and children [13]. Quantification of sTfR and serum ferritin also enables the use of Cook's equation [5,14] to calculate total body iron stores (i.e., mg/kg), a minimally-invasive proxy measure of the gold standard [5] definition of total body stores using bone marrow aspirate [15,16]. In this context, it is important to make tools for quantification of SF and sTfR widely accessible to paediatricians and other health care providers.

Several approaches have been introduced to quantify sTfR: binding assays with radiolabelled transferrin [17,18], ELISA-based assays [19,20], and automated immunoturbidimetric methods [21] (IDeA sTfR-IT; Orion Diagnostica) on a 7600 analyzer (Hitachi). However, many settings lack access to centralized laboratories required for these diagnostic methods. At present, there are no commercially available portable point-of-care testing technologies for assessment of iron status assessment at home or in field locations.

Lateral flow immunoassay (LFIA) [22], commonly referred to as test strips, is a paper-based platform for detection and quantification of analytes in complex samples. Application of LFIAs has expanded over the years to multiple fields in which rapid tests are required as they are low-cost, mass-producible, disposable, equipment free, and require no external power to operate [22]. Recently, advances in smartphone app development have facilitated the use of mobile devices for portable diagnostics [23,24], personal health monitoring [25], and electronic health record management [26]. There is an urgent need for methods to screen for iron status to help early identification of infants and children at risk for ID by rapid, accurate, and cost-effective methods that are easily accessible in most settings. In this study, we present the development of a immunochromatographic assay-based portable screening method on mobile platform for point-of-care quantification of sTfR from a drop of human serum within a few minutes. We demonstrate quantification of physiologically relevant sTfR levels in standard solutions, commercial calibrators, and small-scale proof-of-concept validation with archived human serum samples for comparing the performance with a commercial ELISA kit.

## 2 Materials and methods

The components of the test strip were selected to achieve optimum flow rates and volume of reagents and sTfR in test samples. Development of the immunoassay involved selection of commercially available antibodies and optimization of their concentrations by iterative method to achieve the required detection limits within the physiological range. The entire testing process is guided by a mobile app, which provides step-by-step instructions to the user. Briefly, the testing process requires a drop of serum sample on the test strip to initiate the test. The camera within the reader captures the relative intensity changes of the coloured bands on the test strip for post-processing by the app to determine the sTfR concentrations. The test strip design was optimized, and calibration curves were determined experimentally with sTfR-spiked standards, commercially available sTfR calibrators (‘Access’, Beckman Coulter, Inc.), followed by small-scale validation with archived serum samples to compare with Ramco ELISA kit. The following sections describe the development process in detail.

### 2.1 Reagents and materials

Gold nanoparticles (InnovaCoat 20OD, 40 nm diameter) were obtained from Expedeon, Inc. High purity sTfR from human plasma and monoclonal mouse anti-human-sTfR antibodies (Hytest Cat# 4Tr26-23D10, RRID: AB_1613124 and Hytest Cat# 4Tr26-13E4, RRID: AB_1613123) were purchased from HyTest Ltd. (Finland). Rabbit anti-Mouse-antibodies (Jackson ImmunoResearch Labs Cat# 315-005-045, RRID: AB_2340038) was purchased from Jackson ImmunoResearch, Inc. (West Grove, PA, USA). Access sTfR calibrators (Cat# A32494) were purchased from Beckman Coulter, Inc. Phosphate buffer saline (PBS) (1×, pH 7.4), Tween 20, bovine serum albumin (BSA), Tris buffered saline (TBS), borate buffer, and sucrose were purchased from Sigma-Aldrich, Inc. All test strip materials including conjugate pad, membrane card (HF180), and cellulose fiber pad were obtained from EMD-Millipore. Sample pad consisting of blood filtration membrane was acquired from mdi Membrane Technologies, Inc. Soluble transferrin receptor-Ramco ELISA kit (Cat# TFC-94) was obtained from Ramco Laboratories (Stafford, TX, USA).

### 2.2 Equipment

The following equipment was required for use in this study: automated lateral flow reagent dispenser (Claremont BioSolutions, Upland, CA) and Chemyx Fusion 200 syringe pump (Claremont BioSolutions LLC), Guillotine cutter (Dahle North America, Peterborough, NH), and Synergy 2 Multi-Mode Microplate Reader (BioTek, Winooski, VT).

### 2.3 AuNP-anti-human-sTfR-antibody conjugate pad preparation

The monoclonal anti-human-sTfR-antibody was coupled with gold nanoparticles (AuNPs) as per the instructions provided in the kit. To remove any excess unbound antibodies, a 1:10 dilution of the quencher with water was added with 10 times the volume of the conjugate mixture, and the suspension was centrifuged at 9000*g* for 10 min. The remaining pellet of AuNP-anti-human-sTfR-antibody conjugates were resuspended in a solution consisting 1:10 dilution of quencher with water. The final optical density (O.D.) was measured using Spectramax 384 at 530 nm. The AuNP-anti-human-sTfR-antibody conjugates were diluted to 0·3 OD in conjugate buffer (2 mM borate buffer with 5% sucrose). The diluted conjugates were applied to the conjugate pad and oven dried at 37 °C for three hours and maintained at room temperature overnight.

### 2.4 Test strip assembly

**Fig. 1:**
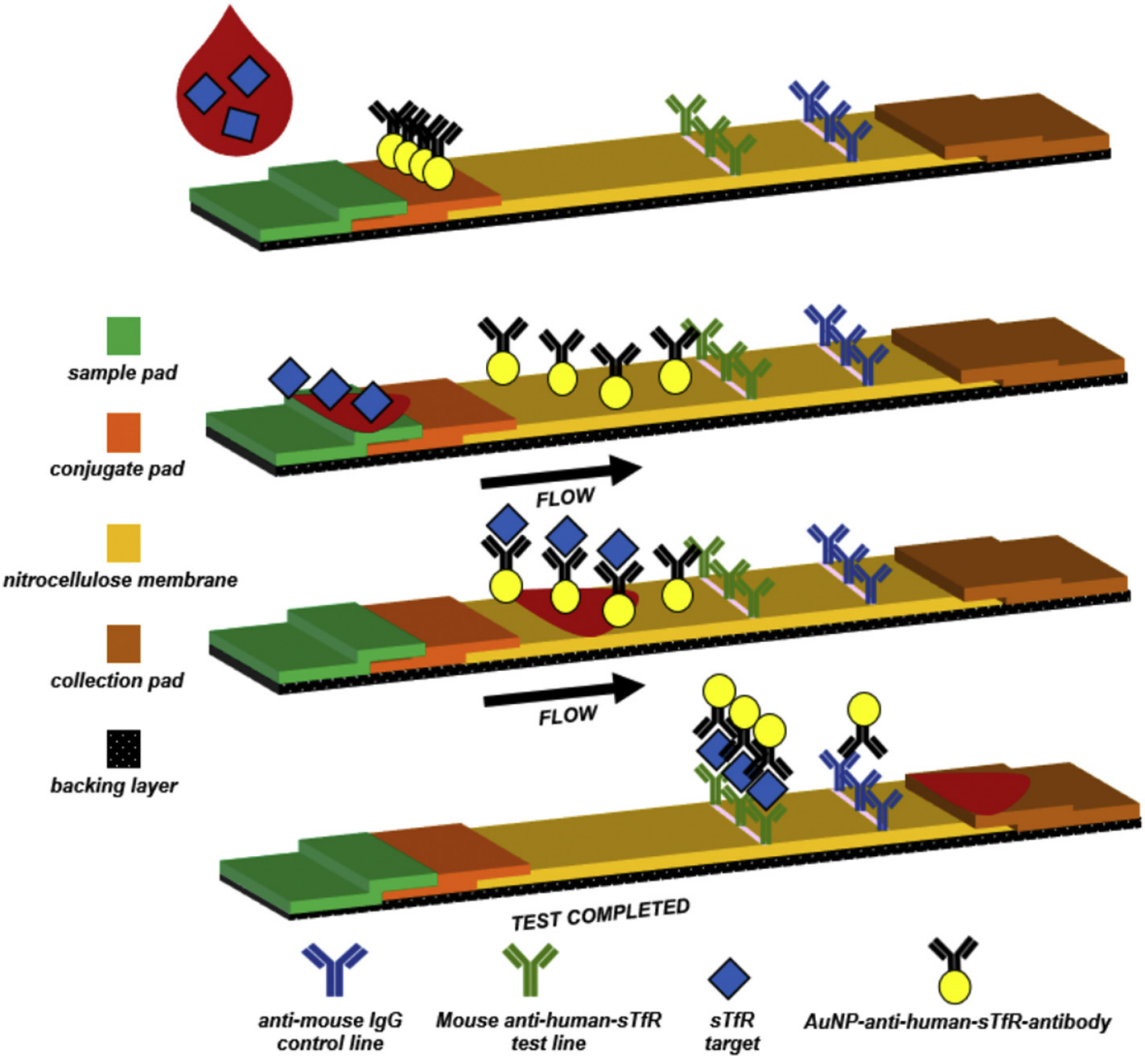
Schematic showing various components of test strip with a sandwich-type assay for sTfR detection.

The membrane card consists of a polyester film backing with nitrocellulose layer on top. Striping of the test and control line antibodies (1 mm wide and 3 mm spacing) consisting of anti-human-sTfR-antibody and anti-mouse-IgG on the nitrocellulose membrane was done using the lateral flow antibody dispenser. Membrane cards were then immediately dried for two hours at 37 °C in forced convection oven and stored at room temperature in a humidity-controlled box. The conjugate pad, absorbent pad, and the sample pad were then assembled with a 2 mm overlap between each pad. The assembled card was cut to obtain test strips of 5 mm width using a guillotine paper cutter. The various components of the sTfR test strip and schematic for a sandwich-type immunoassay are shown in Fig. 1.

### 2.5 Technology and components

The technology consists of a custom developed sTfR test strip, cassette for housing the test strip, test strip reader, and a mobile app for guiding the user through the various steps of the testing protocol. Image processing component of the mobile app is applied to compute the test (T) and control (C) line intensity ratios (T/C) to predict sTfR concentration based on a calibration curve.

### 2.6 Test strip configuration and immunoassay scheme

The sTfR test strip (Fig. 1) was based on a sandwich-format immunoassay detection and consists of whole blood filtration membrane as the sample pad, a conjugate pad for pre-storing the AuNP-anti-human-sTfR-antibody conjugates in dry form, a nitrocellulose membrane with anti-human-sTfR monoclonal antibodies and secondary antibodies, respectively, and wicking/absorbent pad made of cellulose fiber that functions as a waste reservoir. The addition of test sample and running buffer causes the AuNP-anti-human-sTfR-antibody conjugates to flow freely due to capillary action and to react with sTfR in the test sample. At high sTfR concentrations in the test sample, most of the AuNP-anti-human-sTfR-antibody conjugates will bind with the free sTfR, to eventually bind to the anti-human-sTfR-antibody on the test line, resulting in a sandwich complex. All of the unbound AuNP-anti-human-sTfR are captured at the control line. This relative binding of the AuNP-human-sTfR -antibody at the test and control lines increases the test line (T) to control (C) line intensity ratio (T/C) in test samples with higher sTfR concentration. Similarly, in test samples with lower sTfR concentrations, binding of the AuNP-anti-human-sTfR-antibody to form a sandwich complex at the test line is reduced, thereby causing an overall decrease in the T/C value.

### 2.7 Testing protocol

**Fig. 2:**
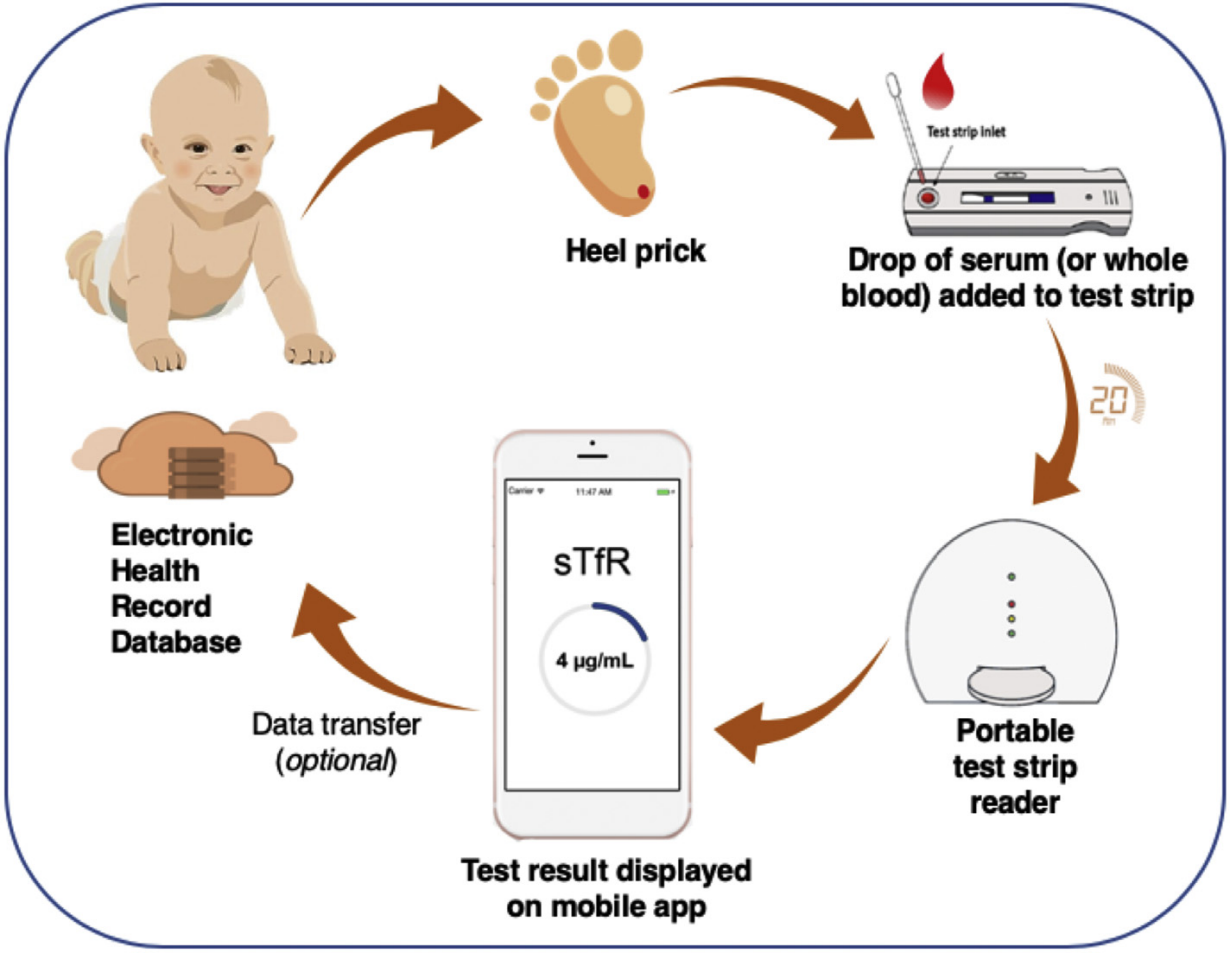
Technology components and testing protocol.

Fig. 2 shows a schematic of the various steps involved in conducting the point-of-care sTfR testing. The user is aided by the step-by-step instructions on the mobile app. Briefly, the user first adds the test sample to test strip, then adds a drop of the chase buffer (1× TBS with 1% BSA, 1·5% Tween20, 0·1% sodium azide) to initiate a capillary flow within the test strip which causes the AuNP-anti-human-sTfR-antibody conjugates to be released from the conjugate pad. The free sTfR in the test sample reacts with AuNP-anti-human-sTfR-antibody and flows downstream to further react with antibodies at the test and control lines. The leftover sample is finally collected in the absorbent pad. The user inserts the test strip into the test strip reader for capturing the colorimetric signals by the camera and analysis by the mobile app to provide the sTfR concentrations.

### 2.8 Preparation of standards

The sTfR-spiked standards in buffer were prepared by serial dilution of purified human sTfR to obtain concentrations in the range of 1 to 10 μg/mL. The Access sTfR calibrators (S0-S5) were available in solution form as provided by the supplier with concentrations ranging from 0 to 12·7 μg/mL.

### 2.9 Image processing algorithm

The mobile app performs image processing steps on the captured test strip image to improve the accuracy and detection limit. Details of the image processing approach have been reported previously [27]. Briefly, captured images are cropped and converted to grayscale to extract the local minima of pixel intensities and calculate the ratio of test to control line intensity (T/C).

## 3 Results

### 3.1 Calibration curve for sTfR-spiked buffer

**Fig. 3:**
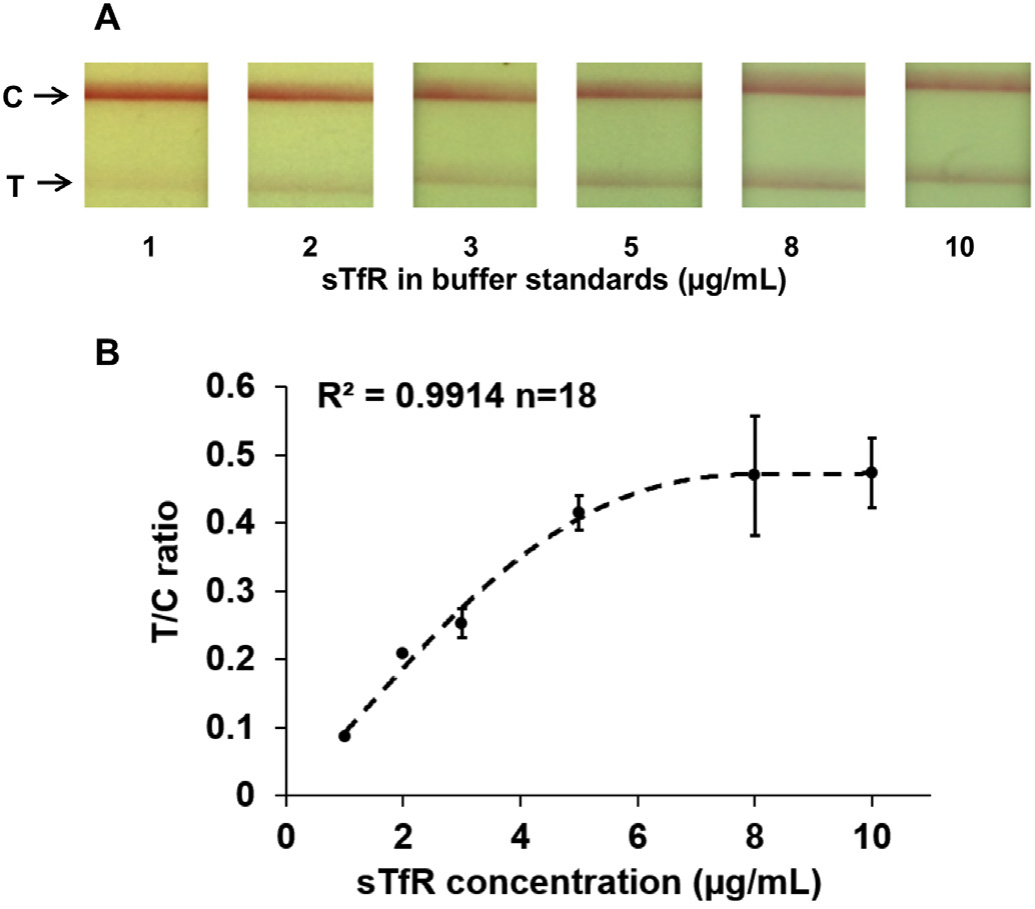
(A) Representative images of the test and control lines on the sTfR test strip for a range of sTfR concentrations in spiked-buffer standards (B) Calibration curve with T/C values for a range of sTfR concentrations in spiked-buffer standards.

Test samples were prepared by diluting stock solution of purified sTfR in standard 1× PBS solution to obtain concentrations in the range 1 to 10 μg/mL. Testing for each concentration was done simultaneously in triplicate. Representative images indicating colorimetric change of the test and control lines on the test strips at various known concentrations of sTfR standards in buffer are presented in Fig. 3A. Test line intensity increases proportionately with increasing sTfR concentrations, as expected for a sandwich-type immunoassay on test strip. For instance, T/C at sTfR concentration of 5 μg/mL was approximately five times higher when compared to T/C at 1 μg/mL. The calibration curve for sTfR-spiked buffer is shown in Fig. 3B; T/C ratios were correlated with the known sTfR standard concentrations. This calibration curve was fitted to derive a function T/C = 0·0001*[sTfR]^4^  – 0·0022*[sTfR]^3^  + 0·0067*[sTfR]^2^  + 0·0886*[sTfR] – 0·0012 where [sTfR] represents sTfR concentration, with an R^2^ value of 0·99. Test results with sTfR-spiked buffer samples confirmed that the selected antibodies provided the required detection limits within the physiological range.

### 3.2 Calibration curve for ‘access’ sTfR calibrators

**Fig. 4:**
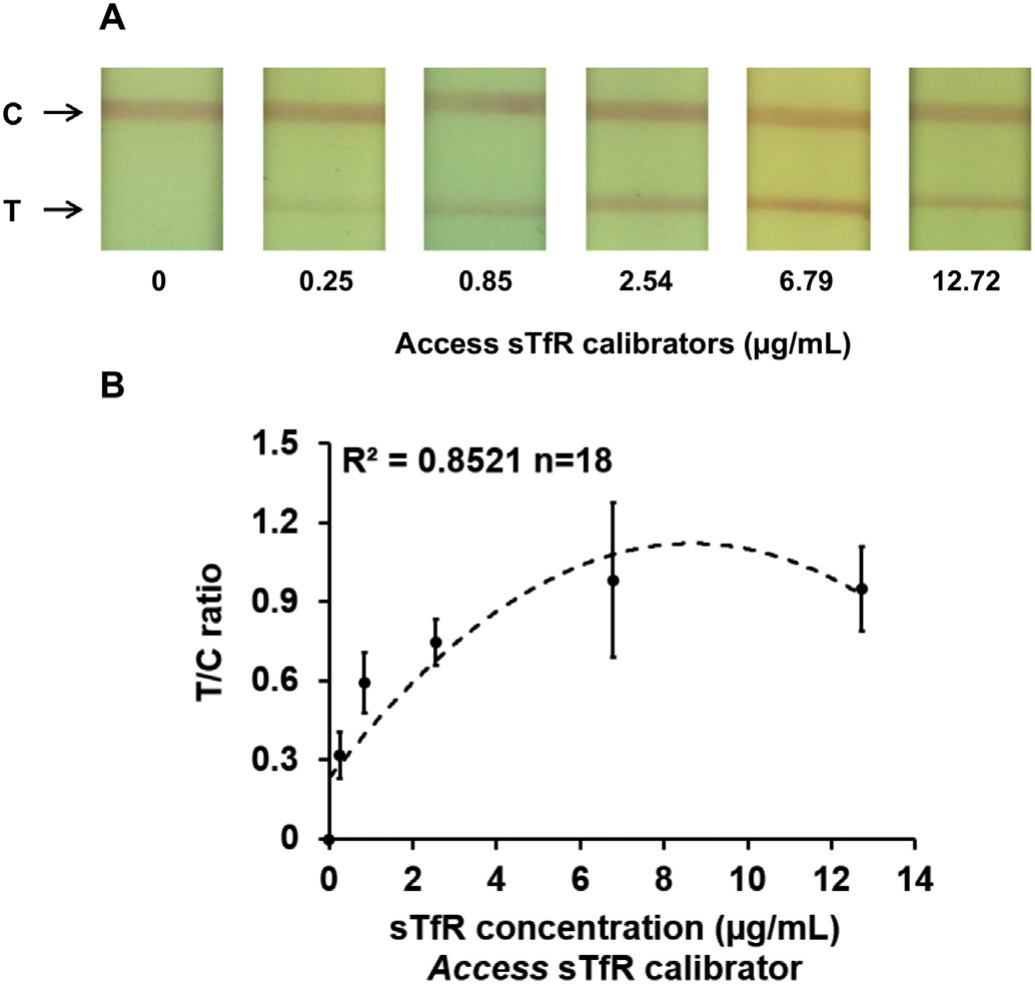
(A) Representative images of the test and control lines on the sTfR test strip for a range of sTfR concentrations of in ‘Access’ calibrators (B) Calibration curve with T/C values for a range of sTfR concentrations of in ‘Access’ calibrators.

Commercially available ‘Access’ sTfR calibrators were obtained as six separate vials labelled S0 to S5 with concentrations ranging from 0 to 12·7 μg/mL. For each concentration of the calibrator, testing was performed in triplicate. Representative images of the test and control lines for each of the known concentrations of ‘Access’ calibrators are presented in Fig. 4A. The calibration curve (Fig. 4B) demonstrates that T/C values were correlated with the sTfR concentrations. The T/C values increased with sTfR concentrations until approximately 9 μg/mL, and then began to decrease beyond physiological range, due to a hook effect [28,29]. The calibration curve was fitted with a second order polynomial to derive a function T/C = −0·0118*[sTfR]^2^  + 0·2054* [sTfR] + 0·2299, where [sTfR] represents sTfR concentration, with an R^2^ value of 0·85 when the curve was fit for the concentration range 0 to 12·7 μg/mL and an R^2^ value of 0·89 when upper concentration limit of the curve was set to 6·8 μg/mL.

### 3.3 Calibration curve for human serum samples

The performance of sTfR test strips was further evaluated with archived human serum samples. Serum samples included commercially available serum samples with known sTfR concentrations provided by the vendor based on Siemens BN II Nephelometer testing. We also included archived serum samples with known ferritin concentrations from our previous study. The inflammation status of these archived serum samples was not available. We used Ramco ELISA kit to determine the sTfR concentrations for both commercially obtained samples and archived serum samples.

**Fig. 5:**
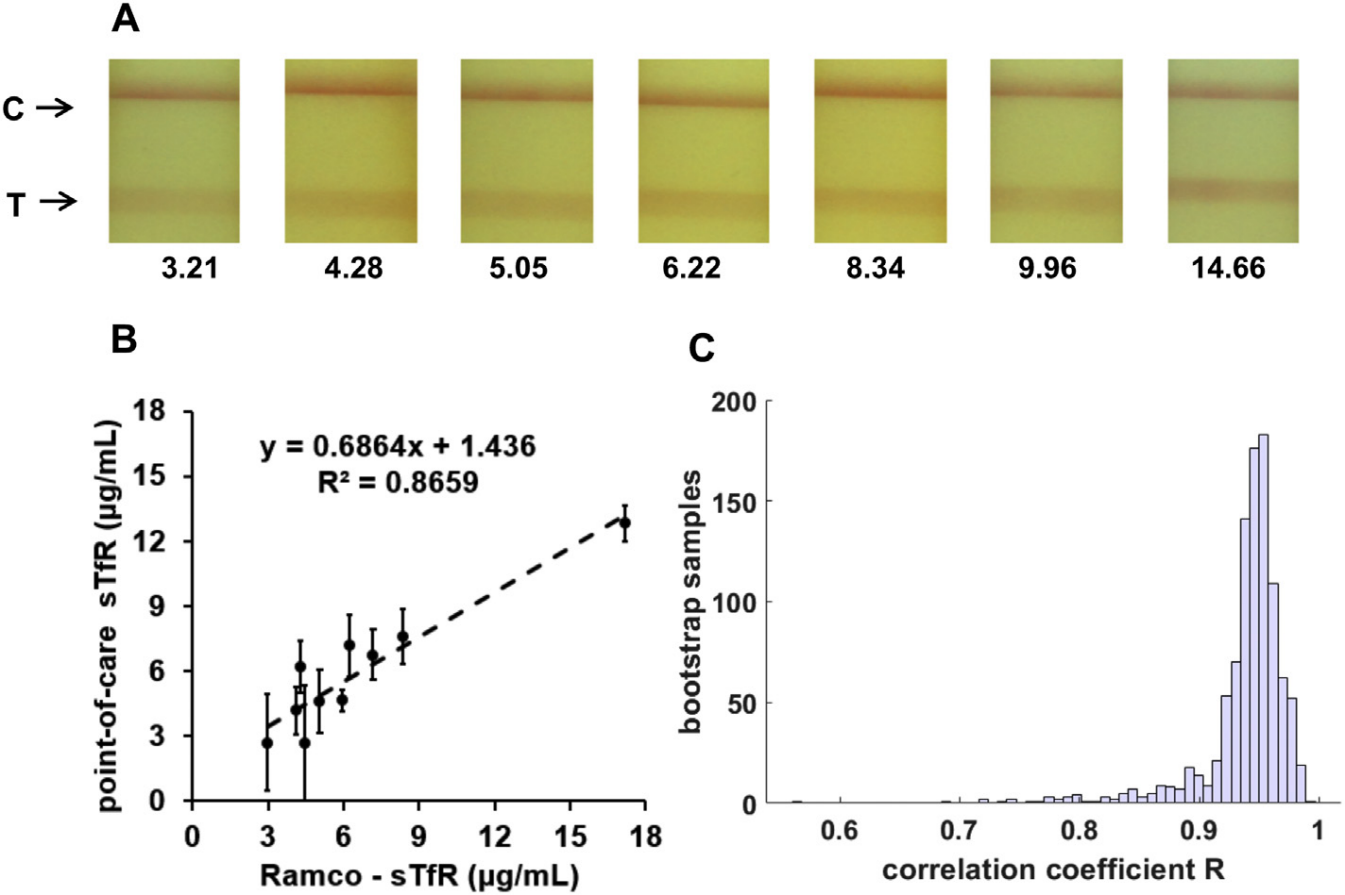
(A) Representative images of the test and control lines on the sTfR test strip for a range of concentrations of serum samples (B) Correlation plot of predicted serum sTfR concentrations based on comparison of point-of-care test results with Ramco ELISA (C) Results of bootstrapping to compute the resulting correlation coefficients.

Fig. 5A shows representative images of test and control lines for a range of selected sTfR concentrations of serum samples. We selected T/C data for six samples and compared our point-of-care sTfR results against the Ramco ELISA test results. A calibration curve with function sTfR_Ramco_ = 44·36*(T/C) – 9·01 was obtained where sTfR_Ramco_ refers to sTfR concentration determined from ELISA kit. Based on this calibration curve the sTfR concentrations of the remaining samples tested by the point-of-care technology was predicted. Fig. 5B shows a correlation plot comparing the sTfR levels predicted by our point-of-care technology against the corresponding levels quantified by Ramco ELISA kit. Results demonstrated a correlation of 0·93 (P < 0.0001), with an R^2^ value of 0·87 with linear fitting applied on the trendline.

Bootstrap resampling analysis was performed using MATLAB with serum test results of 16 samples to assess correlations between T/C ratios determined on point-of-care system with Ramco-based sTfR concentrations. The bootstrapping function was applied to resample 1000 times and the resulting correlation coefficients were computed. Fig. 5C presents the results of bootstrapping in a histogram, which indicates that most of the correlation coefficient estimates lie on the interval [0·8 1·0]. A 95% confidence interval for the correlation coefficient between T/C ratios and sTfR concentrations of the tested serum samples was also obtained [0·60 0·96].

Findings from bootstrap analyses provide quantitative evidence that T/C ratios from our point-of-care technology and sTfR concentrations are highly correlated.

In our previous work on development of lateral flow assay for ferritin, ferritin concentrations were quantified for samples used in the present study. Cook's equation was used to calculate total body iron (TBI), using ferritin and sTfR values [14].

$$\mathrm{TBI}\;\left(\mathrm{mg}/\mathrm{kg}\right)=-\left[\log_{10}\;\left[\mathrm{sTfR}\left(\mathrm{mg}/L\right)\times 1000/\mathrm{SF}\left(\mathrm{\mu g}/L\right)\right]–2\cdot 8229\right]/0\cdot 1207$$

**Fig. 6:**
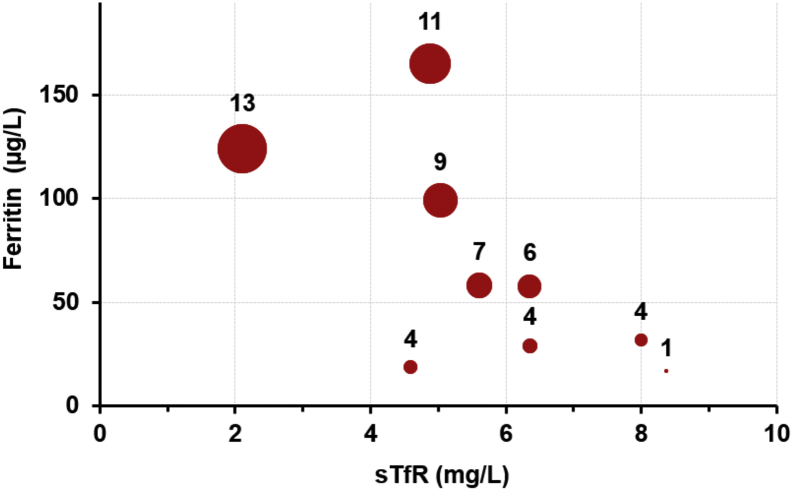
Total body iron estimation based on Cook's equation with known sTfR and ferritin concentrations.

The results are presented in the form of a bubble plot in Fig. 6. In analyses using the archived serum samples, most samples had sTfR concentrations below the Ramco recommended cut-off level (>8·3 μg/mL). Our point-of-care sTfR screening method on mobile platform has a sensitivity and specificity of 100% and 83.3% respectively for a cut-off at 6 μg/mL.

## 4 Discussion

There is an ongoing debate on the application of the current AAP recommendation of universal screening for iron deficiency through measurement of haemoglobin level at 1 year of age. One of the concerns is that Hb lacks sensitivity and specificity for ID since the levels overlap between iron sufficiency and deficiency, and there are other causes of anaemia besides ID. There is a need for better methods at the point-of-care for screening and assessment of iron deficiency among infants and young children. Another major concern in case of infants and young children with a time sensitive and critical phase where their brain is rapidly developing, is that there is a risk of brain becoming iron deficient before the onset and detection of anaemia. At present there is a lack of consensus on which iron status variables to use for infants in a multiple criteria model [30]. The risk of ID affecting neurodevelopment by the time anaemia is detected makes a stronger case for testing at 12 months of age for ID than anaemia [31]. The timing and methods used for screening for ID in infants and young children are controversial and further studies are required to generate evidence on this subject [4].

Assessment of iron status with tests such as serum ferritin may be a more promising screening test for ID in childhood. Beyond ferritin assessment alone, quantification of sTfR is a critical step for improved differential diagnosis of ID anaemia. Serum sTfR and ferritin quantification can be applied to Cook's equation for total body iron estimation. At present, in most settings, measurement of ferritin and sTfR levels requires access to centralized laboratories by using blood obtained by venepuncture. Central laboratories have the advantage of highly trained technicians who are skilled at operating automated immunoanalyzers capable of high-throughput testing with high sensitivity and specificity. However, this approach is time consuming and not cost-effective. In the current scenario with uncertainties regarding screening strategy and the need for further data for new strategies, it is essential for paediatricians and healthcare workers to have easy and affordable access to point-of-care diagnostics for assessment of ID. Point-of-care testing can be an effective screening tool by providing much faster access to test results that allows for more rapid clinical decision making and timely intervention. However, the reliability of testing could be affected by lack of sufficient training in personnel and when testing conditions and protocols are not strictly followed.

In this study, we demonstrated a rapid, user-friendly sandwich-type test strip on our mobile platform for quantification of sTfR levels in human serum samples. We determined calibration curves for the sTfR assay on mobile platform with sTfR standards in buffer and commercially available ‘Access’ sTfR calibrators, followed by small-scale proof-of-concept validation with archived serum samples. The detection range demonstrated in buffer and ‘Access’ sTfR calibrator was 1 to 8 μg/mL. The sTfR assay on mobile platform was successfully applied to quantify sTfR from archived human serum samples, and the performance was comparable to Ramco ELISA kit, a widely accepted method for sTfR detection. The reference range of the assay based on calibration with Ramco ELISA kit is 2–18 μg/mL with sTfR concentration > 8·3 μg/mL considered as ID. Further, the mobile device component of this technology can enable easy communication of test results by e-mail or text message, with option to upload to electronic health record databases. This data processing enables personal, confidential communication of health information as well as a foundation for population-level surveillance.

In future studies, the performance of the point-of-care sTfR screening method on mobile platform will need to be assessed using serum or whole blood in human validation studies among greater number of participants with a wider range of iron status including iron deficient individuals for a more rigorous evaluation of diagnostic test accuracy. Human validation studies will enable broader performance analysis of the point-of-care sTfR diagnostic tool in real-time. Future studies will also evaluate a duplex assay for measurement of sTfR and ferritin on a single test strip, facilitating iron status assessment at the point-of-care. A duplex approach will also inform the estimation of total body iron using Cook's equation, as a non-invasive proxy measure for the gold standard definition of body iron stores using bone marrow aspirate [15,16].

We developed and validated a point-of-care sTfR screening method on our mobile platform for quantifying serum sTfR concentrations from a drop of human serum. Based on the preliminary testing results, the point-of-care sTfR screening device reported in this study signifies a critical step towards highly sensitive home-use test kits and in both clinical and field locations for real-time sTfR quantification. Our future work will focus on developing a duplex assay on a single test strip to perform simultaneous quantification of sTfR and ferritin to enable estimation of total body iron. Dual assessment of both iron status biomarkers on a point-of-care mobile platform has the potential to transform diagnostics and inform interventions for ID prevention globally.
